# Effectiveness of a whole health model of care emphasizing complementary and integrative health on reducing opioid use among patients with chronic pain

**DOI:** 10.1186/s12913-022-08388-2

**Published:** 2022-08-17

**Authors:** Steven B. Zeliadt, Jamie H. Douglas, Hannah Gelman, Scott Coggeshall, Stephanie L. Taylor, Benjamin Kligler, Barbara G. Bokhour

**Affiliations:** 1 VA Center of Innovation (COIN) for Veteran-Centered and Value-Driven Care, VA Puget Sound Healthcare System, 1660 South Columbian Way, HSR&D S-152, Seattle, WA 98108 USA; 2 Department of Health Services, School of Public Health, University of Washington, 1660 South Columbian Way, HSR&D S-152, Seattle, WA 98108 USA; 3 Center for the Study of Healthcare Innovation, Implementation and Policy, Greater Los Angeles VA Healthcare System, Los Angeles, CA USA; 4 Department of General Internal Medicine and Department of Health Policy and Management, UCLA, Los Angeles, CA USA; 5 Department of Family and Community Medicine, Icahn School of Medicine at Mount Sinai, Brooklyn, NY USA; 6 US Department of Veterans Affairs Office of Patient Centered Care and Cultural Transformation, Washington, DC USA; 7 Center for Healthcare Organization and Implementation Research, VA Bedford Healthcare System, Bedford, MA USA; 8 Department of Population and Quantitative Health Sciences, University of Massachusetts Medical School, Worcester, MA USA

**Keywords:** Opioid analgesics, Pain management, Whole Health, Complementary and Integrative health, Complementary and Alternative medicine, Veterans

## Abstract

**Background:**

The opioid crisis has necessitated new approaches to managing chronic pain. The Veterans Health Administration (VHA) Whole Health model of care, with its focus on patient empowerment and emphasis on nonpharmacological approaches to pain management, is a promising strategy for reducing patients’ use of opioids. We aim to assess whether the VHA’s Whole Health pilot program impacted longitudinal patterns of opioid utilization among patients with chronic musculoskeletal pain.

**Methods:**

A cohort of 4,869 Veterans with chronic pain engaging in Whole Health services was compared with a cohort of 118,888 Veterans receiving conventional care. All patients were continuously enrolled in VHA care from 10/2017 through 3/2019 at the 18 VHA medical centers participating in the pilot program. Inverse probability of treatment weighting and multivariate analyses were used to adjust for observable differences in patient characteristics between exposures and conventional care. Patients exposed to Whole Health services were offered nine complementary and integrative health therapies alone or in combination with novel Whole Health services including goal-setting clinical encounters, Whole Health coaching, and personal health planning.

**Main measures:**

The main measure was change over an 18-month period in prescribed opioid doses starting from the six-month period prior to qualifying exposure.

**Results:**

Prescribed opioid doses decreased by -12.0% in one year among Veterans who began complementary and integrative health therapies compared to similar Veterans who used conventional care; -4.4% among Veterans who used only Whole Health services such as goal setting and coaching compared to conventional care, and -8.5% among Veterans who used both complementary and integrative health therapies combined with Whole Health services compared to conventional care.

**Conclusions:**

VHA’s Whole Health national pilot program was associated with greater reductions in prescribed opioid doses compared to secular trends associated with conventional care, especially when Veterans were connected with complementary and integrative health therapies.

**Supplementary Information:**

The online version contains supplementary material available at 10.1186/s12913-022-08388-2.

## Background

As part of the 2016 Comprehensive Addiction and Recovery Act [1], the Veterans Health Administration (VHA) embarked on a large demonstration project to pilot the Whole Health model of care in 18 medical centers across the country [2]. The implementation of Whole Health at these sites was specifically intended to help reduce opioid use. In the past decade, there has been a call to transform how pain is treated in response to increases in dependence on opioids and a substantial rise in opioid-related deaths [3, 4]. Although treatment guidelines now emphasize the use of non-pharmacological treatments [5–7], and while there is an extensive evidence base related to individual complementary and integrative health (CIH) therapies and pain management [8–13], there is limited evidence showing the effectiveness of health system efforts to deliver these therapies broadly, and evidence showing the effectiveness of a Whole Health model of care is even more limited [14].

**Fig. 1:**
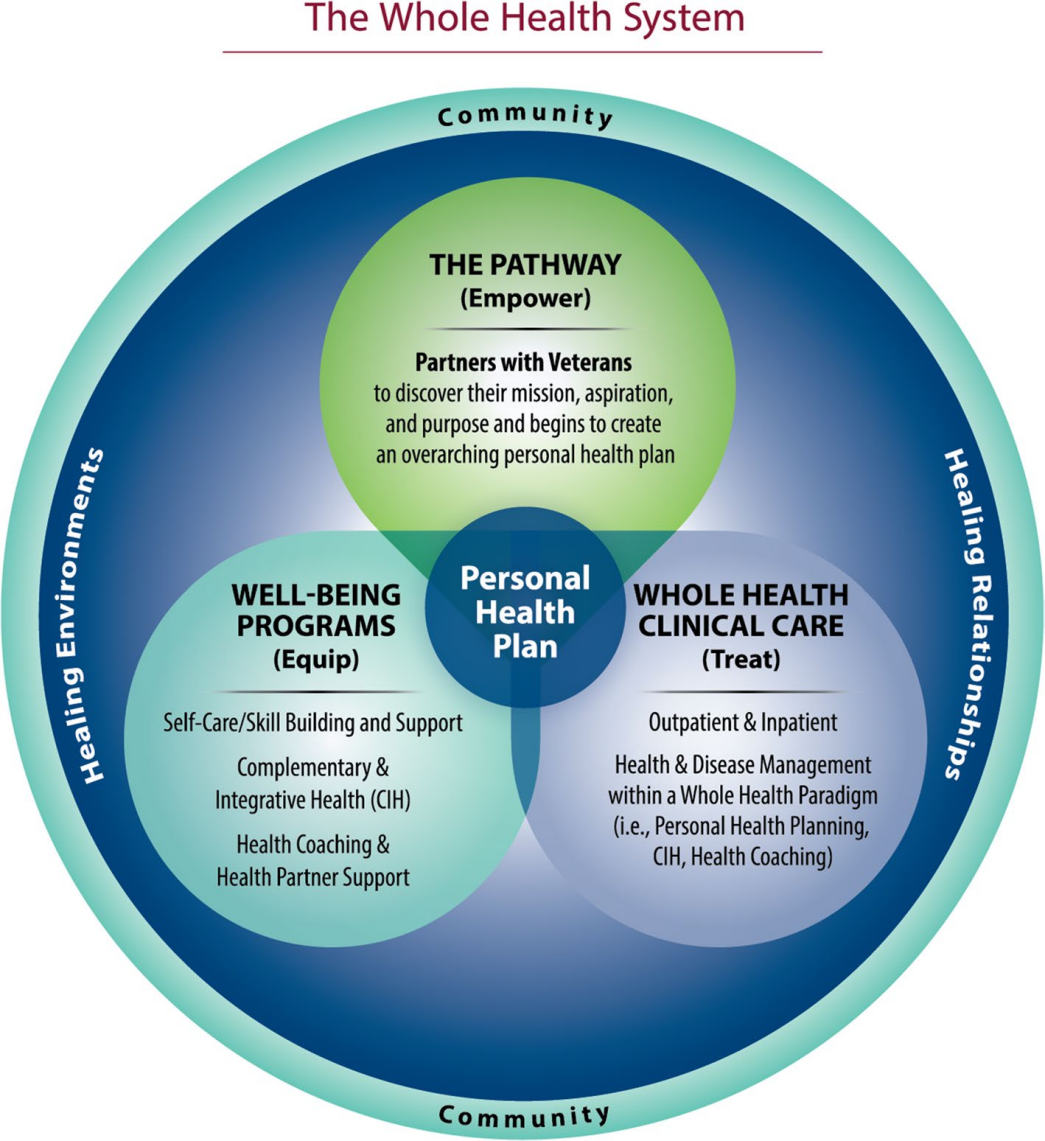
VHA’s whole health model of care

The VHA-developed Whole Health model of care takes an interdisciplinary approach emphasizing non-pharmacologic pain management therapies, self-care, skill building, and support, that moves from a medical/disease-oriented system to one that also focuses on well-being. The Whole Health System of Care has three main components (Fig. 1): 1) Pathway in which Veterans explore and identify their personal health goals; 2) Well-being programs, which include CIH approaches, Whole Health coaching, and educational classes which equip patients with skills for self-care; 3) Clinical care in which clinicians collaborate with patients to provide care that aligns with each individual’s personal health goals. For many patients with chronic pain, Whole Health often involves referral to CIH therapies that have a focus on mind, body and self-care skills related to pain management.

The objective of this evaluation is to assess how VHA’s pilot Whole Health program impacted prescription opioid use. We focus on Veterans’ utilization of two sets of activities: 1) CIH therapies, and 2) Whole Health services including goal-setting, personal health planning and coaching. We assess how these activities, either alone or in combination, affected Veterans’ use of opioids for chronic pain management.

## Methods

This evaluation was conducted as part of a congressionally-mandated effort to assess VHA’s Whole Health pilot program included in the Comprehensive Addiction and Recovery Act (CARA) of 2016 (Public Law No:114–198) [1]. The findings presented in this manuscript were derived from this non-research operations activity in accordance with VHA Handbook 1058.05 and Program Guide 1200.21. We report the findings according to the Strengthening the Reporting of Observational Studies in Epidemiology (STROBE) reporting guideline.

### Study design and study population

**Fig. 2:**
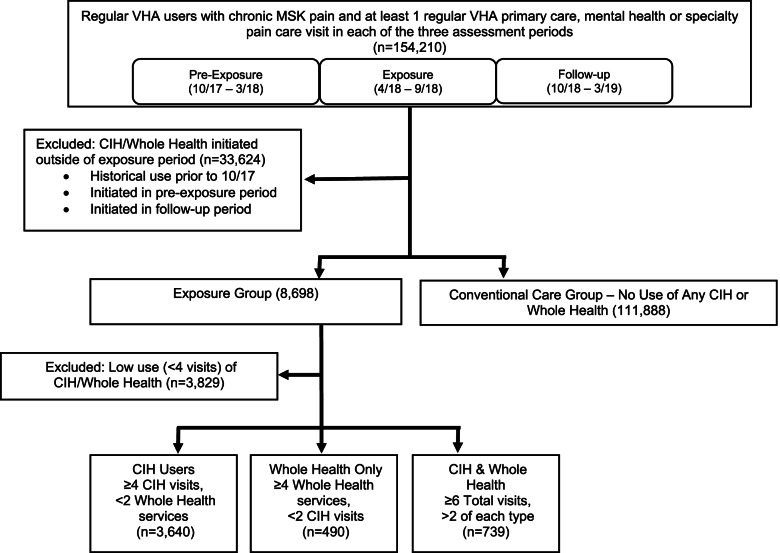
Consort diagram

Using the VHA’s Corporate Data Warehouse, we identified Veterans with chronic musculoskeletal pain at the 18 VHA medical centers participating in the Whole Health pilot program. The assessment periods consisted of six-month/two-quarter blocks starting with the initiation of the pilot program and are labeled as Pre-Exposure (10/1/2017–3/31/2018), Exposure (4/1/2018–9/30/2019) and Follow-Up (10/1/2019–3/31/2019) (Fig. 2). The exposure period started 4/1/2018 to allow the pilot sites time to hire CIH therapy providers and initiate programs, and corresponded with an expansion of Whole Health services [15]. Patients were included if they were regular users of healthcare, defined as having at least one visit during each of the three study periods. Chronic musculoskeletal pain was identified during the pre-exposure study period using an electronic health record (EHR) algorithm based on timing and accumulation of ICD10 codes [16, 17].

### Exposures

Veterans participated in Whole Health and CIH through a variety of referral and recruitment methods across the 18 pilot sites including development of referral order sets, internal marketing and awareness efforts within clinics, and flyers, advertising, Facebook, website calendars and other outreach efforts directly to Veterans. Referral to Whole Health and CIH varies by clinic and is influenced by many factors including underlying PCP and pain team practices, as well as the aforementioned marketing and awareness efforts, especially when new providers are hired. Three levels of exposure were determined a priori. The three exposure groups were defined as 1) patients who utilized a combination of Whole Health services and additional CIH therapies; 2) patients who received only Whole Health services, and 3) patients who used only CIH therapies. Although the VA considers chiropractic care to be conventional medical care, it was included in the analysis as a CIH therapy. A comparison group termed “conventional care” was constructed by identifying Veterans receiving care at the 18 pilot sites who met the chronic pain eligibility criteria but did not utilize any Whole Health services or CIH therapies during the study period. Exposure was determined by total accumulated visits during the exposure and follow up periods. Patients with at least one but fewer than four visits to any combination of CIH therapies or Whole Health services were not considered exposed, yet were also not considered unexposed, and thus were excluded from all analysis (*n* = *3,829*). All patients with chronic pain, including those not using opioids in the pre-exposure period, were included in the study population so that possible longitudinal effects of CIH therapies and Whole Health services on the initiation of opioids were captured.

We used both structured coding methods (e.g., CPT codes, VHA accounting codes) and semi-structured methods (e.g., clinic note titles) to identify CIH therapies and Whole Health services [15]. CIH therapies included acupuncture, chiropractic care, therapeutic massage, yoga, Tai Chi/Qigong, meditation, guided imagery, clinical hypnosis, and biofeedback. Whole Health services included Whole Health Pathway, Whole Health education/skills classes, personal health planning, and Whole Health coaching. We also used VHA community care claims data to identify acupuncture, therapeutic massage and chiropractic care paid for by the VHA but performed by community providers [18]. Detailed methods of specific codes and how they were identified and combined are now publicly available [19, 20].

### Opioid outcomes

Opioid prescriptions were extracted from VHA’s Pharmacy Managerial Cost Accounting National Data Extract. All results are reported as the average mg morphine equivalent (MME) per patient per day averaged over each quarter (MME/patient-quarter). Because patients in all exposure groups had to continuously use VA throughout all study periods and were covered over the full quarterly periods, this can be converted to average daily or monthly dose. Secondary analyses focused on changes in average quarterly dose for different levels of pre-exposure dose. By using pre-exposure dose, and not dose during the same period as initial exposure to Whole Health or CIH, longitudinal change in the dose outcome is less likely to be due to regression to the mean. Opioid prescriptions were converted into MME using the Centers for Disease Control and Prevention Opioid definitions based on drug name, dosage, and quantity [21]. Opioid prescriptions were identified using the VHA drug class code CN101 excluding fills for injectable fentanyl as well as buprenorphine and non-tabular forms of methadone (liquid, solution, and injectable). Only outpatient prescription data sources were utilized. Outliers above 95% within each drug name dose were replaced with the 95% value. Negative quantities (assumed to represent returns) were rare and included as they appeared in the data.

### Covariates

We examined additional characteristics calculated from the period prior to utilizing any CIH therapies or Whole Health services to adjust for potential confounding biases. These demographic and clinical characteristics included the type of chronic musculoskeletal pain calculated based on the start of the exposure study period, common chronic medical and mental health conditions, and variables related to socioeconomic status including rurality and level of copayments required by VHA based on service-connected injuries and income means test [22].

### Analysis

Data were analyzed between 7/2020 and 3/2021. Using descriptive statistics, the three exposure groups were compared to patients with chronic musculoskeletal pain who used conventional healthcare services without use of any Whole Health services or CIH therapies. Unpaired t-tests were used to compare continuous variables, and chi-square tests were used to compare categorical variables across the exposure groups.

We used propensity score analysis with inverse probability to treatment weighting (IPTW) to identify populations of Veterans among the conventional healthcare users who were as comparable as possible to each of the three exposure groups. Three separate IPTW logistic regression models were constructed corresponding to each of the three exposure groups. All three models compared patients using Whole Health and/or CIH to the larger group of conventional care patients (*n* = 111,888). For this, we used available observable patient and clinical characteristics (Table 1). Patients’ facility was included as a fixed effect to account for the variation in availability of CIH therapies across sites. IPTW models were estimated using pre-exposure data only, before any patient included in the study received any CIH therapies or Whole Health services. Balance in patient and clinical characteristics was assessed by calculating standardized differences between the weighted groups using graphical methods (Additional file 1) [23]. Covariate balance for the model examining propensity to use only Whole Health services was improved by not including facility. The R *evalue *package was used to estimate the effect of potential unmeasured confounders [24].

**Table 1:** Baseline demographics and clinical characteristics of patients receiving conventional care compared to patients using CIH therapies and Whole Health services

	Conventional Care	Complementary and Integrative Health (CIH) Only	Whole Health Only	CIH and Whole Health	*p*-value
Counts of Users, No	111,888	3,640	490	739	–
Starting Quarterly Morphine Equivalent, mean (SD), mg	628.5 (2,286.9)	689.9 (2,555.8)	465.1 (1,771.0)	670.9 (2,336.7)	0.151
Any Opioid Use, No. (%)	35,520 (31.7)	1,224 (33.6)	121 (24.7)	253 (34.2)	< 0.001
Long Term Opioid Treatment at Baseline	18,612 (16.6)	625 (17.2)	55 (11.2)	107 (14.5)	0.004
Type of Chronic Pain, No. (%)	–	–	–	–	–
Multiple	60,268 (53.9)	2,918 (80.2)	328 (66.9)	626 (84.7)	< 0.001
Back	12,946 (11.6)	349 (9.6)	38 (7.8)	23 (3.1)	
Limb/Extremity	25,600 (22.9)	200 (5.5)	85 (17.3)	40 (5.4)	
Chest	3,268 (2.9)	10 (0.3)	7 (1.4)	6 (0.8)	
Neck	1,436 (1.3)	25 (0.7)	4 (0.8)	3 (0.4)	
Headache, Fibromyalgia, and Other Conditions	2,071 (1.9)	27 (0.7)	7 (1.4)	17 (2.3)	
Pain identified during pre-exposure sampling but did not meet EHR definition at baseline	6,299 (5.6)	111 (3.0)	21 (4.3)	24 (3.2)	
Chronic and Mental Health Conditions, No. (%)	–	–	–	–	–
Rheumatoid Arthritis	4,111 (3.7)	170 (4.7)	23 (4.7)	36 (4.9)	0.003
Cancer	1,714 (1.5)	41 (1.1)	11 (2.2)	6 (0.8)	0.044
Cardiovascular Disease	30,236 (27.0)	757 (20.8)	139 (28.4)	175 (23.7)	< 0.001
Hypertension	72,907 (65.2)	2,009 (55.2)	346 (70.6)	457 (61.8)	
Diabetes	40,837 (36.5)	1,072 (29.5)	224 (45.7)	278 (37.6)	
Obesity	25,050 (22.4)	928 (25.5)	257 (52.4)	308 (41.7)	
COPD	24,110 (21.5)	665 (18.3)	110 (22.4)	143 (19.4)	
PTSD	34,183 (30.6)	1,346 (37.0)	172 (35.1)	289 (39.1)	
Depression	34,184 (30.6)	1,376 (37.8)	240 (49.0)	392 (53.0)	
Anxiety	21,380 (19.1)	949 (26.1)	139 (28.4)	220 (29.8)	
Alcohol Use Disorder	12,250 (10.9)	417 (11.5)	83 (16.9)	113 (15.3)	
Psychosis	3,522 (3.1)	100 (2.7)	17 (3.5)	31 (4.2)	0.192
Female, No. (%)	11,915 (10.6)	611 (16.8)	93 (19.0)	164 (22.2)	< 0.001
Age, No. (%)	–	–	–	–	–
18–39	9,748 (8.7)	561 (15.4)	40 (8.2)	68 (9.2)	< 0.001
40–49	11,242 (10.0)	604 (16.6)	56 (11.4)	111 (15.0)	
50–59	21,427 (19.2)	860 (23.6)	135 (27.6)	231 (31.3)	
60–69	36,142 (32.3)	932 (25.6)	164 (33.5)	213 (28.8)	
70–79	26,750 (23.9)	582 (16.0)	88 (18.0)	105 (14.2)	
80–90	6,579 (5.9)	101 (2.8)	7 (1.4)	11 (1.5)	
Race, No. (%)	–	–	–	–	–
Black	26,823 (24.0)	665 (18.3)	192 (39.2)	264 (35.7)	< 0.001
White	76,950 (68.8)	2,692 (74.0)	272 (55.5)	442 (59.8)	
Other	2,579 (2.3)	122 (3.4)	10 (2.0)	13 (1.8)	
Not Reported	5,536 (4.9)	161 (4.4)	16 (3.3)	20 (2.7)	
Hispanic Ethnicity, No. (%)	–	–	–	–	–
Non-Hispanic	100,780 (90.1)	3,309 (90.9)	443 (90.4)	679 (91.9)	< 0.001
Hispanic	6,897 (6.2)	208 (5.7)	40 (8.2)	48 (6.5)	
Not Reported	4,211 (3.8)	123 (3.4)	7 (1.4)	12 (1.6)	
Marital Status, No. (%)	–	–	–	–	–
Married	64,544 (57.7)	2,113 (58.0)	253 (51.6)	398 (53.9)	0.061
Not Married	46,717 (41.8)	1,508 (41.4)	234 (47.8)	337 (45.6)	
Not Reported	627 (0.6)	19 (0.5)	3 (0.6)	4 (0.5)	
VA Service Connection, No. (%)	–	–	–	–	–
100%	23,958 (21.4)	981 (27.0)	111 (22.7)	210 (28.4)	< 0.001
50–99%	39,946 (35.7)	1,502 (41.3)	204 (41.6)	330 (44.7)	
< 50%	19,135 (17.1)	552 (15.2)	81 (16.5)	107 (14.5)	
Not Connected	28,849 (25.8)	605 (16.6)	94 (19.2)	92 (12.4)	
Residential Location, No. (%)	–	–	–	–	–
Rural	25,744 (23.0)	853 (23.4)	51 (10.4)	108 (14.6)	< 0.001
Urban	86,144 (77.0)	2,787 (76.6)	439 (89.6)	631 (85.4)	

Fully adjusted generalized linear models of change in prescription opioid doses from the pre-exposure to post-exposure period were then estimated using the IPTW weights from the propensity models including the demographic and clinical characteristics to account for additional residual confounding. Facility was included as a random effect to account for variation in prescription opioid patterns across the 18 pilot locations and potential variation in underlying availability of services across the sites. Summary estimates of the adjusted amount of change and adjusted percent change in opioid dose attributable to exposure to each of the integrative health treatments relative to usual care were calculated holding all covariates constant relative to weights of usual care patients using margins in R which utilizes a delta method approach.

## Results

A total of 4,869 Veterans with chronic musculoskeletal pain initiating CIH therapies and novel patient-centered Whole Health services between 10/2017 and 9/2019 were identified at 18 VHA medical centers participating in the Whole Health pilot. We also identified a comparison cohort of 111,888 Veterans with chronic pain receiving only traditional care with no exposure to Whole Health or CIH therapies who continuously used VHA care during the same period at the same facilities. The majority of exposed patients, 3,640, used only CIH therapies, while 490 used only Whole Health services, and 739 engaged both CIH therapies and Whole Health services.

The patient characteristics of each group before adjustment are described in Table 1. Patients using conventional care differed from those using CIH therapies and/or Whole Health services. Conventional care users were more likely to be older, male, had more physical health problems outside of chronic musculoskeletal pain and fewer psychological health conditions. Conventional care patients were also slightly less likely to be on opioid treatment during the pre-exposure period compared to Veterans who started using CIH therapies, although Veterans who started using only Whole Health services were the least likely to be on an opioid treatment during the pre-exposure period. In unadjusted analyses, average morphine dose in the pre-exposure period was lowest among patients who went on to start using only Whole Health services—465 MME/quarter. Initial levels during the pre-exposure period varied slightly across patients who used conventional care (628 MME/quarter), patients who used CIH therapies (690 MME/quarter), and patients who used both CIH therapies and Whole Health services (671 MME/quarter).

After weighting, there were few differences in patient demographic and clinical characteristics between the three exposure groups and each group’s matched conventional care comparison group (Table 2). Differences in use of opioid treatment remained across the groups during the pre-exposure period, a variable that was intentionally not included in the propensity score weighting. Use varied from 31.8% among the conventional care group, 34.8% among patients who used CIH therapies only (*p* = 0.018), 24.5% among patients who used Whole Health services only (*p* = 0.011), and 34.1% among patients who used both CIH therapies and Whole Health services (*p* = 0.475). We present only the covariate distributions for the conventional care group weighted to match the group of Veterans who used CIH therapies only; although, the columns of p-values represent comparisons to each exposure group’s respective weighted conventional care group.

**Table 2:** Baseline demographics and clinical characteristics after adjustment with IPTW weights

	Weighted –Conventional Care^a^	Weighted –Complementary and Integrative Health (CIH) Only	*p*-value	Weighted – Whole Health Only	*p*-value	Weighted – CIH and Whole Health	*p*-value
Counts of Users, No	111,888	3,640	–	490	–	739	–
Starting Quarterly Morphine Equivalent, mean (SD), mg	630.6 (2,286.4)	700.3 (2,694.2)	0.278	567.5 (1,823.9)	0.575	674.2 (2,076.5)	0.756
Any Opioid Use, %	31.8	34.8	0.018	24.5	0.011	34.1	0.475
Type of Chronic Pain, %	–	–	–	–	–	–	–
None	5.6	6.1	0.053	5.9	0.780	7.7	0.164
Multiple	54.7	58.0		56.3		62.9	
Back	11.5	12.7		12.3		8.7	
Limb/Extremity	22.3	18.7		19.9		15.8	
Chest	2.8	1.8		3.7		2.9	
Neck	1.3	1.0		0.6		0.7	
Headache, Fibromyalgia, and Other Conditions	1.8	1.7		1.4		1.3	
Chronic and Mental Health Conditions, %	–	–	–	–	–	–	–
Rheumatoid Arthritis	3.7	3.7	0.981	4.5	0.500	3.8	0.894
Cancer	1.5	1.4	0.683	1.0	0.227	1.3	0.789
Cardiovascular Disease	26.8	26.6	0.833	24.1	0.272	30.2	0.322
Hypertension	64.9	65.9	0.394	64.3	0.766	64.6	0.868
Diabetes	36.3	35.9	0.792	36.1	0.871	34.2	0.509
Obesity	22.5	24.4	0.095	25.3	0.156	28.4	0.042
COPD	21.5	22.8	0.230	18.5	0.214	23.2	0.605
PTSD	30.7	31.6	0.496	35.9	0.070	32.8	0.480
Depression	30.8	32.5	0.162	37.8	0.009	32.1	0.620
Anxiety	19.3	20.5	0.247	22.1	0.183	22.5	0.199
Alcohol Use Disorder	11.0	10.5	0.550	12.5	0.342	12.3	0.551
Psychosis	3.1	2.9	0.642	3.7	0.622	3.0	0.882
Female, %	10.8	11.6	0.285	12.8	0.199	13.3	0.148
Age, %	–	–	–	–	–	–	–
18–39	8.9	8.2	0.738	10.2	0.221	8.8	0.888
40–49	10.2	9.8		12.8		10.8	
50–59	19.3	20.4		22.7		20.6	
60–69	32.1	32.4		26.9		33.4	
70–79	23.7	23.9		23.7		22.1	
80–90	5.8	5.3		3.7		4.2	
Race, %	–	–	–	–	–	–	–
Black	23.8	23.0	0.659	25.2	0.523	27.0	0.086
White	68.9	70.3		68.2		64.9	
Other	2.3	2.4		3.1		0.8	
Not Reported	4.9	4.4		3.5		7.3	
Hispanic Ethnicity, %	–	–	–		–		–
Non-Hispanic	90.1	91.7	0.151	88.7	0.381	92.7	0.282
Hispanic	6.1	5.2		8.3		5.0	
Not Reported	3.8	3.1		2.9		2.2	
Marital Status, %	–	–	–	–	–	–	–
Married	57.7	59.8	0.077	57.0	0.622	53.3	0.326
Not Married	41.7	39.9		42.0		45.8	
Not Reported	0.6	0.3		1.1		0.9	
VA Service Connection, %	–	–	–	–	–	–	–
100%	21.6	22.3	0.749	25.2	0.287	22.6	0.939
50–99%	35.9	36.1		35.9		36.6	
< 50%	17.0	16.0		17.7		16.0	
Not Connected	25.5	25.5		21.2		24.8	
Residential Location, %	–	–	–	–	–	–	–
Rural	23.0	24.8	0.113	19.9	0.340	19.4	0.267
Urban	77.0	75.2		80.1		80.6	

^a^Only one of the three weighted Conventional Care groups – weighted to match CIH users only – is presented as weighted distributions for all three groups were similar

In unadjusted analyses of all groups, including the conventional care group, all experienced a substantial decrease in opioid dose over the 18-month period, which was consistent with national VHA trends (Table 3). The unadjusted decrease in prescription opioid doses was -12.5% (628 MME to 550 MME) for patients who used conventional care only; -24.6% (690 MME to 520 MME) among patients who used CIH therapies only; -19.2% (465 MME to 376 MME) for patients who used Whole Health services only, and -37.4% (671 MME to 420 MME) among patients who used both CIH therapies and Whole Health services. The subset of Veterans who met criteria for long term opioid use at baseline and who had higher starting levels of opioids in the pre-exposure period had larger unadjusted decreases in opioid dose which followed similar trends across the exposure groups: -15.7% (3,422 MME to 2,589 MME), -30.0% (3,663 MME to 2,564 MME), -21.3% (3,745 MME to 2,946 MME), and -49.7% (4,146 MME to 2,084 MME), respectively.

**Table 3:** Changes in prescription opioid doses associated with conventional care and utilization of CIH and Whole Health services

	Conventional Care	CIH Only	Whole Health Only	CIH & Whole Health
Time Trend—Unadjusted Differences in Opioid Use (Overall)				
MME Pre, Mg (SD)	628 (2,287)	690 (2,556)	465 (1,771)	671 (2,337)
MME Post, Mg (SD)	550 (2,116)	520 (1,902)	376 (1,604)	420 (1,903)
MME Difference, Mg (SD)	-78 (1,117)	-170 (1,327)	-89 (666)	-251
% Change	-12.5%	-24.6%	-19.2%	-37.4%
*p*-value	< 0.001	< 0.001	< 0.001	< 0.001
Effect Attributed to CIH/Whole Health compared to Usual Care—Adjusted Differences in Opioid Use (Overall)				
MME Difference, Mg (SD)	n/a	-76 (-86.6 to -64.4)	-28 (-39.7 to -15.4)	-53 (-71.7 to -34.7)
% Change	n/a	-12.0% (-13.8 to -10.2)	-4.4% (-6.3% to -2.4%)	-8.5% (-11.4% to -5.5%)
*p*-value	n/a	< 0.001	< 0.001	< 0.001
e-value	n/a	1.29	1.18	1.17
Adjusted Differences in Opioid Use (Stratified by Initial MME)				
% Change by Pre-Exposure MME Levels				
50^th^ (900 MME)	n/a	-11.8% (-13.0 to -10.5)	-1.6% (-3.0 to -0.3)	-13.0% (-15.1 to -11.0)
75^th^ (2400 MME)	n/a	-12.5% (-13.1 to -12.0)	2.4% (1.8 to 3.0)	-21.7% (-22.6 to -20.7)
95^th^ (7200 MME)	n/a	-12.8% (-13.3 to -12.4)	4.0% (3.5 to 4.5)	-25.1% (-25.9 to -24.3)

In adjusted analyses using conventional care as a comparison group, we estimated the decrease in prescription opioid dose attributable to the three exposures. We observed decreases of -12.0% associated with use of CIH therapies only; -4.4% associated with Whole Health services only; and -8.5% associated with the use of both CIH therapies and Whole Health services (Table 3). E-values, which represent the minimum association an unmeasured confounder would need to have to explain away the observed exposure-outcome association accounting for all other measured confounders, were 1.29, 1.18, and 1.17, respectively, for the three analyses.

We conducted a sensitivity analysis to examine the effect of the three exposures among patients with different starting levels of opioid prescription doses in the pre-exposure period to assess adjusted estimates for patients with higher pre-expose dose levels, such as Veterans meeting long term opioid therapy (LTOT) criteria (Table 3). Among patients who used both CIH therapies and Whole Health services, those with higher starting opioid prescription doses experienced larger decreases compared to the conventional care group. We found mixed results for patients who used Whole Health services only, although the number of patients with high starting opioid prescription doses in this exposure group was sparse.

## Discussion

VHA and other healthcare systems have implemented multiple initiatives to help patients with chronic pain reduce their reliance on opioids. VHA’s demonstration project to pilot the Whole Health model of care and expand availability of CIH therapies in 18 medical centers led many patients to begin using these services, often for the first time. Findings from this evaluation suggest these efforts have helped reduce patients’ reliance on opioids.

It is notable that patients who used CIH therapies, either alone (-12.0%) or in combination with Whole Health services (-8.5%), experienced the greatest reductions in prescription opioid doses. These findings suggest that CIH therapies, which have been demonstrated in effectiveness trials to help manage pain, play an important role in helping reduce reliance on opioids [5–7]. The use of Whole Health services alone was also associated with a meaningful reduction in opioid dose (-4.4%). While this reduction was observed in a small subset of patients and was notably smaller than in Veterans who used CIH therapies either alone or in combination, these findings suggest that a Whole Health-only approach to care may be useful for patients who do not wish to use CIH. This finding is consistent with a prior national VHA evaluation that observed that VHA medical centers with more non-pharmacological pain management offerings, including CIH therapies, had fewer patients initiating long-term opioid therapy [25]. We had anticipated patients using CIH therapies combined with Whole Health services would experience the largest declines because their level of prescription opioid doses declined the most (-37.4%) in the unadjusted data. Adjustment for patient differences attenuated the decreases in prescription opioid dose levels associated with exposure to Whole Health combined with CIH therapies and exposure to Whole Health only. These attenuations in the adjusted analysis do not necessarily suggest that combining CIH with Whole Health is less effective than CIH or Whole Health alone, but are likely due to removing confounding and selection bias present in the unadjusted analysis. Delivery of CIH and Whole Health continue to expand and evolve in VA and these data may not reflect how Whole Health is combined with CIH therapies in the future. One notable change is the pivot to telehealth delivery, with many Whole Health services now being delivered via telehealth [19, 20]. In this analysis CIH therapies were much more widely available and more commonly used. With expansion of Whole Health and telehealth delivery it is possible that Whole Health may become more prevalent both alone and in combination with CIH therapies. Since 2012, opioid use in VHA has decreased by 64% [26], and the demographic groups most likely to use Whole Health services as reflected in Table 1, including younger Veterans and Veterans with depression and other mental health diagnoses, have more overlap with other opioid safety initiatives in VHA. Additionally, while we explored outcomes for the three exposure groups compared with conventional care, the focus of this evaluation was not to directly assess which specific combinations of Whole Health services and CIH therapies are most effective. Instead, we sought to understand if the services as they were offered in the pilot program were associated with reductions in opioid use. Future efforts should continue to replicate these findings in additional populations and explore which specific combinations or individual CIH therapies and Whole Health services are most beneficial.

This study has several strengths, including its use of the Whole Health pilot program as a natural experiment and its national scale. It also has several limitations. Identifying exposure to CIH therapies and Whole Health services through the VHA’s EHR is challenging. Historically, many CIH therapies have not been captured by coding in the VHA’s EHR due to the lack of established CPT codes [27], although a key part of VHA’s demonstration project was to provide standard coding procedures for both CIH therapies and Whole Health services [20]. Patients can participate in CIH therapies outside of the healthcare system, therefore we may have not fully captured utilization of CIH therapies. Because use of CIH therapies and Whole Health services was not randomized, there is the potential for selection bias, with patients opting to engage in these therapies possibly being more motivated in general, and thus more likely to reduce their reliance on opioids regardless of their use of CIH therapies or Whole Health services. Additionally, providers who are initiating conversations about tapering may be more likely to refer or encourage Whole Health and CIH therapies, highlighting the non-causal association of their use with opioid reductions. Additional studies are exploring the importance of offering these therapies when attempting to taper [28]. A similar quasi-experimental study of CIH therapies in VHA found that IPTW methods, similar to methods used in our study, were successful in accounting for confounding bias by eliminating baseline differences across exposure groups [29]. The use of IPTW methods in our study was successful in reducing most baseline differences between the three exposure groups and patients using conventional care (Additional file 1). There is the potential for residual bias that was not fully removed by the IPTW methods, especially among the small exposure group of patients using Whole Health services. Even after weighting, this group had lower levels of opioid use during the pre-exposure period although pre-exposure dose was included as a balancing factor in the IPTW model. The e-values (Table 3) suggest that it is moderately unlikely that unmeasured confounding exists that would explain away the observed relationships [24]. Additionally, while we included both patients who had and did not have initial opioid prescription during the pre-exposure period to follow longitudinally, future studies may want to consider a two-part approach in which the probability of any opioid use is assessed longitudinally, and then evaluate changes in dose among that subset of patients.

## Conclusions

Overall, this evaluation demonstrates the potential value of the VHA and other healthcare systems investing in the Whole Health model of care and expanding the availability of CIH therapies for pain management. VHA’s Whole Health national pilot program was associated with greater reductions in prescribed opioid doses compared to secular trends associated with conventional care, especially when Veterans were connected with CIH therapies. These broad, population-level findings support the growing evidence-based use of individual CIH therapies and components of Whole Health for pain and opioid management, and demonstrate the real-world impact that system change can have on patients.

## Supplementary Information

**Additional file 1.** Balance in Baseline Covariates After Inverse Probability of Treatment Weighting (IPTW).

## Data Availability

The datasets generated and analyzed in this study are derived from data in VHA’s Corporate Data Warehouse. These data are available to individuals who have authorized approval to access VHA medical record data. Code to replicate the findings can be made available to authorized individuals from the corresponding author. We report the findings according to the Strengthening the Reporting of Observational Studies in Epidemiology (STROE) reporting guideline.

## Abbreviations

VHA: Veterans Health Administration
CIH: Complementary and Integrative Health
CARA: Comprehensive Addiction and Recovery Act
STROBE: Strengthening the Reporting of Observational Studies in Epidemiology
EHR: Electronic Health Record
LTOT: Long Term Opioid Therapy
MME: Mg Morphine Equivalent
IPTW: Inverse Probability to Treatment Weighting
