# Supplementary material for: Effectiveness of a whole health model of care emphasizing complementary and integrative health on reducing opioid use among patients with chronic pain
Source: BMC Health Serv Res. 2022 Aug 17;22:1053. doi: 10.1186/s12913-022-08388-2 (PMC9387037; doi:10.1186/s12913-022-08388-2)

Additional file 1. Balance in Baseline Covariates After Inverse Probability of Treatment Weighting (IPTW)


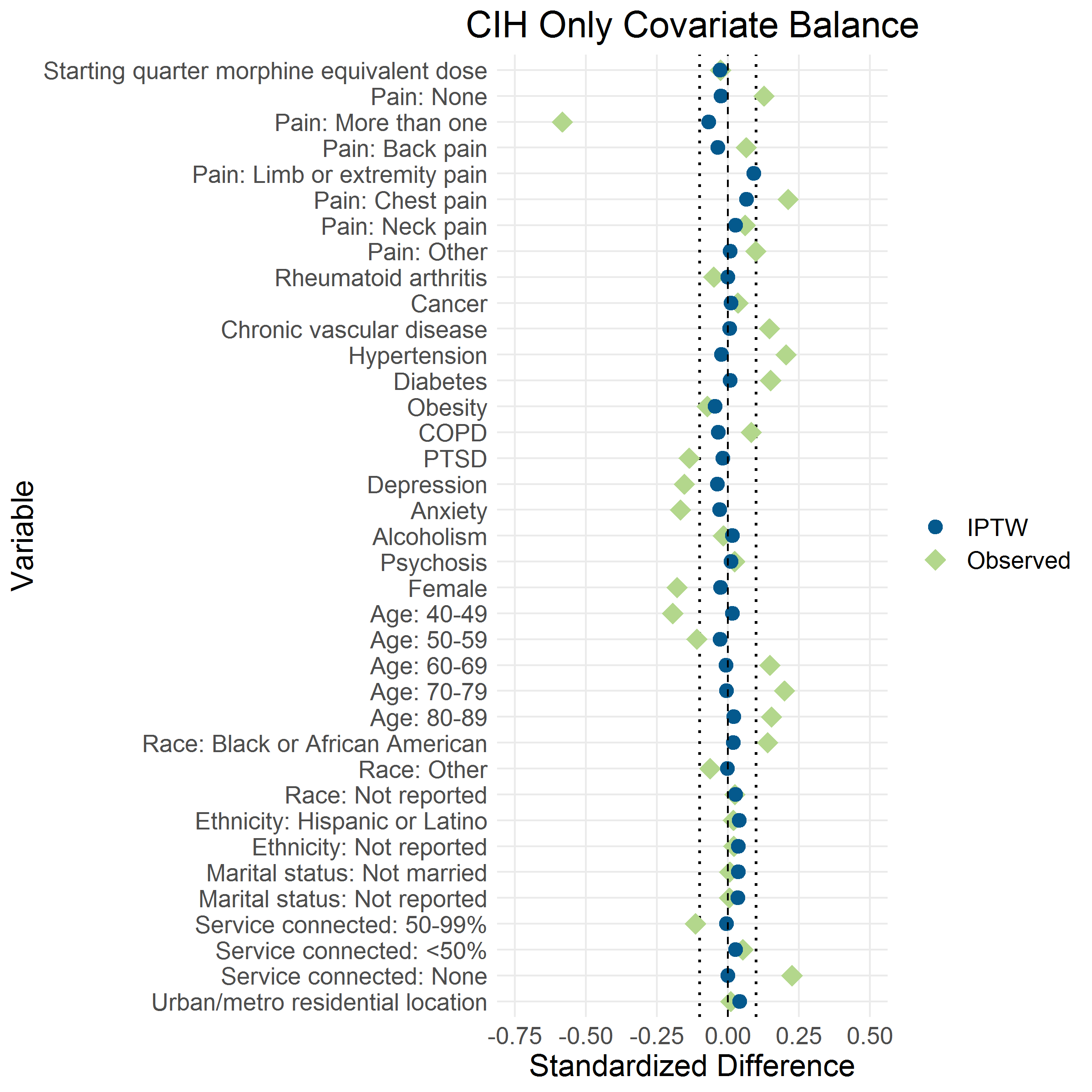


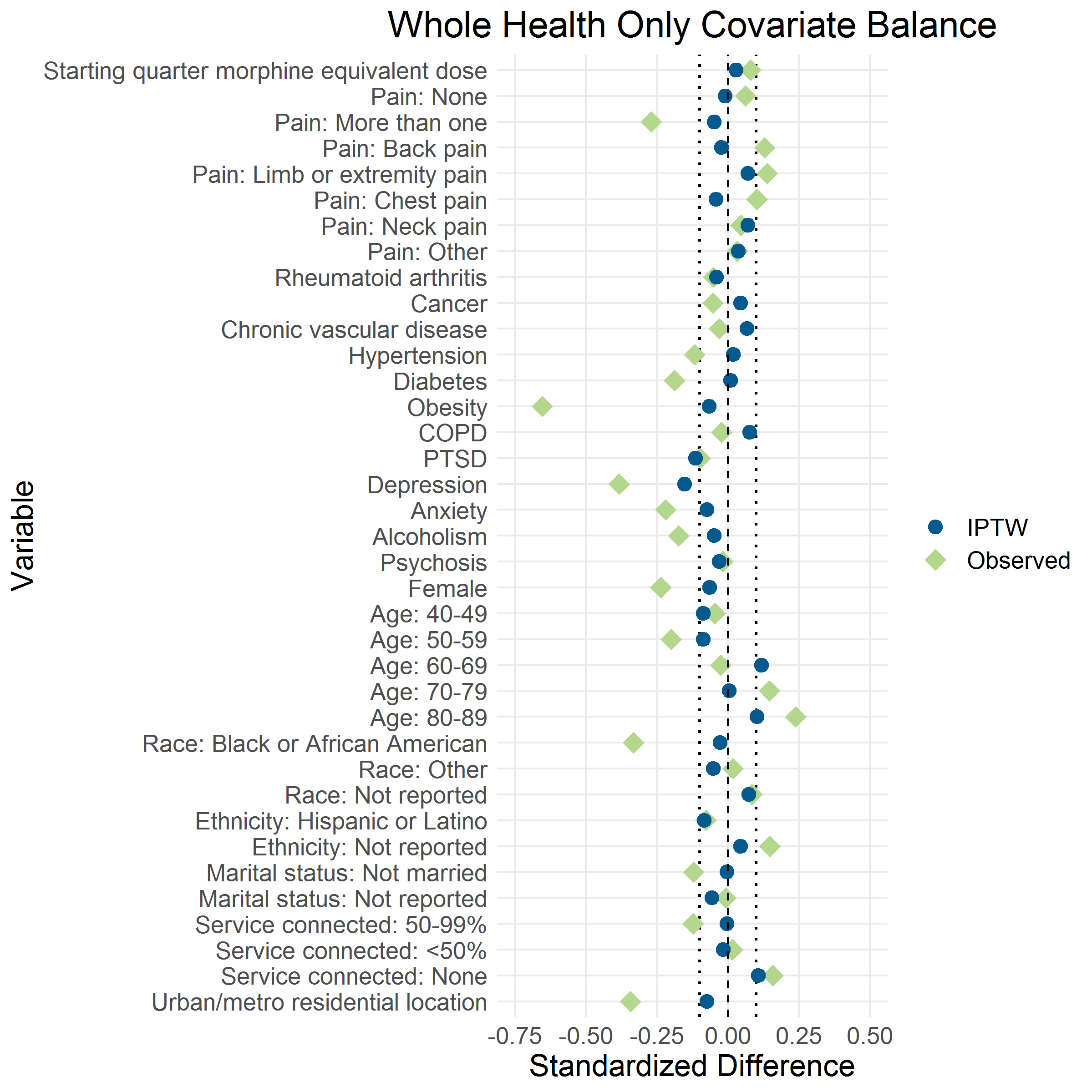


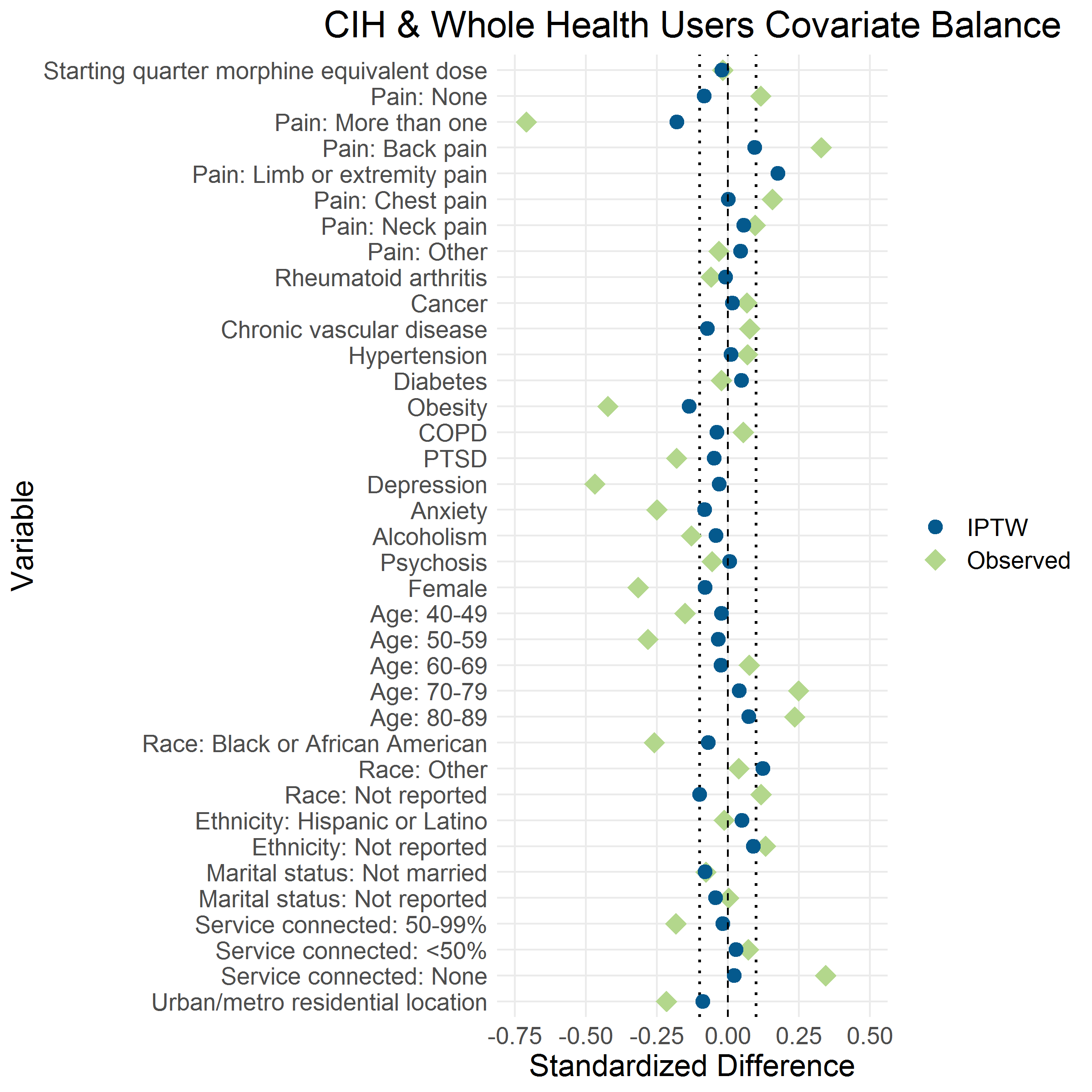

Supplement: Supplementary file 1 — Additional file 1. Balance in Baseline Covariates After Inverse Probability of Treatment Weighting (IPTW). [file 12913_2022_8388_MOESM1_ESM.docx]
